# Structural rearrangements generate cell-specific, gene-independent CRISPR-Cas9 loss of fitness effects

**DOI:** 10.1186/s13059-019-1637-z

**Published:** 2019-02-05

**Authors:** Emanuel Gonçalves, Fiona M. Behan, Sandra Louzada, Damien Arnol, Euan A. Stronach, Fengtang Yang, Kosuke Yusa, Oliver Stegle, Francesco Iorio, Mathew J. Garnett

**Affiliations:** 1 Wellcome Sanger Institute, Wellcome Genome Campus, Hinxton, UK; 2 European Molecular Biology Laboratory, European Bioinformatics Institute, Hinxton, UK; 3 GSK, Medicines Research Center, Gunnels Wood Road, Stevenage, SG1 2NY UK

**Keywords:** CRISPR, Structural rearrangements, Ploidy, Copy-number, Crispy

## Abstract

**Background:**

CRISPR-Cas9 genome editing is widely used to study gene function, from basic biology to biomedical research. Structural rearrangements are a ubiquitous feature of cancer cells and their impact on the functional consequences of CRISPR-Cas9 gene-editing has not yet been assessed.

**Results:**

Utilizing CRISPR-Cas9 knockout screens for 250 cancer cell lines, we demonstrate that targeting structurally rearranged regions, in particular tandem or interspersed amplifications, is highly detrimental to cellular fitness in a gene-independent manner. In contrast, amplifications caused by whole chromosomal duplication have little to no impact on fitness. This effect is cell line specific and dependent on the ploidy status. We devise a copy-number ratio metric that substantially improves the detection of gene-independent cell fitness effects in CRISPR-Cas9 screens. Furthermore, we develop a computational tool, called Crispy, to account for these effects on a single sample basis and provide corrected gene fitness effects.

**Conclusion:**

Our analysis demonstrates the importance of structural rearrangements in mediating the effect of CRISPR-Cas9-induced DNA damage, with implications for the use of CRISPR-Cas9 gene-editing in cancer cells.

**Electronic supplementary material:**

The online version of this article (10.1186/s13059-019-1637-z) contains supplementary material, which is available to authorized users.

## Background

Genetic loss-of-function screens are used to systematically identify genes important for cellular fitness and genetic interactions in model organisms [1, 2]. Traditionally, these have been performed with RNA interference (RNAi) [3–5], although its application to mammalian cells has been hampered by incomplete protein depletion and off-target effects [6, 7]. The advent of CRISPR-Cas technologies facilitates gene editing of human cells by addressing many of the limitations of RNAi and increases capacity to identify genes essential for cellular fitness [8–13]. In cancer cell lines, CRISPR-Cas9 dropout screens have been integrated with genomic data sets to propose novel therapeutic targets [3, 14–16]. Tumor cell genetic instability can induce synthetic-lethal dependencies on genes that otherwise have no impact on cellular fitness [17].

Gene copy-number changes, despite being rare and often detrimental in normal cells [18], are one of the most frequent types of genomic alterations in cancers [19]. They are of particular importance when analyzing CRISPR-Cas9 experiments because targeting genomic regions that are copy-number amplified induces DNA damage responses that lead to cell cycle arrest and cell death [20, 21]. The effect is gene-independent and ubiquitous across cancer types. This increases the false-positive rate of gene loss of fitness (LOF) detection when interpreting results using CRISPR-Cas9 reagents targeting amplified regions. We and others have developed computational methods to account for this systematic bias [22, 23]. Some of these approaches are guided by knowledge of gene copy-number values, which on average are proportional to the non-specific LOF effect of CRISPR-Cas9 targeting. Nonetheless, the strength of this association varies between cell lines and amplicons with similar copy-number and is completely absent in some cases [23]. This indicates that other cellular features besides copy-number influence non-specific CRISPR-Cas9 LOF effects.

Cancer cells undergo extensive genomic alterations [24–26] and the impact of these on response to CRISPR-Cas9 targeting is poorly understood. Here, we combined CRISPR-Cas9 screens with whole-genome sequencing (WGS) and DNA SNP6 copy-number arrays to investigate the impact of structural variation (SV) on CRISPR-Cas9 response. We find that LOF effects of CRISPR-Cas9 screens mediated by copy-number amplifications are associated with structural rearrangements such as tandem duplications, in stark contrast to amplifications arising from chromosomal duplication. Considering this, we show that gene copy-number ratios, normalized by chromosome copy-number, provide more accurate identification of gene independent LOF effects. Additionally, we provide a novel computational tool that takes these effects into consideration on a per sample basis to provide accurate measurements of CRISPR-Cas9 LOF effects.

## Results

### Increased cell ploidy buffers non-specific CRISPR-Cas9 LOF effects

We considered publicly available genome-wide CRISPR-Cas9 knockout screens (BROAD DepMap 18Q3 depmap.org/portal/) performed in 36 different tumor types comprising 250 cancer cell lines [22, 27], which have been previously genomically characterized for copy-number and gene expression [28, 29] (Additional file 1: Figure S1a, Additional file 2: Table S1). Gene-essentiality fold-change profiles were estimated for a total of 17,328 genes, each targeted on average by 3.8 single-guide RNAs (sgRNAs). For the majority of the cell lines, two technical replicates were performed and gene averaged log-fold change values had a mean Pearson correlation (*R*) of 0.79. Genes previously defined as essential for cellular viability [15] were robustly recapitulated in all samples (mean area under recall curve (AURC) = 0.86), and as previously described [21], non-detrimental genes displayed a small enrichment for positive fold changes (mean AURC = 0.42) (Additional file 1: Figure S1b and S1c).

**Fig. 1:**
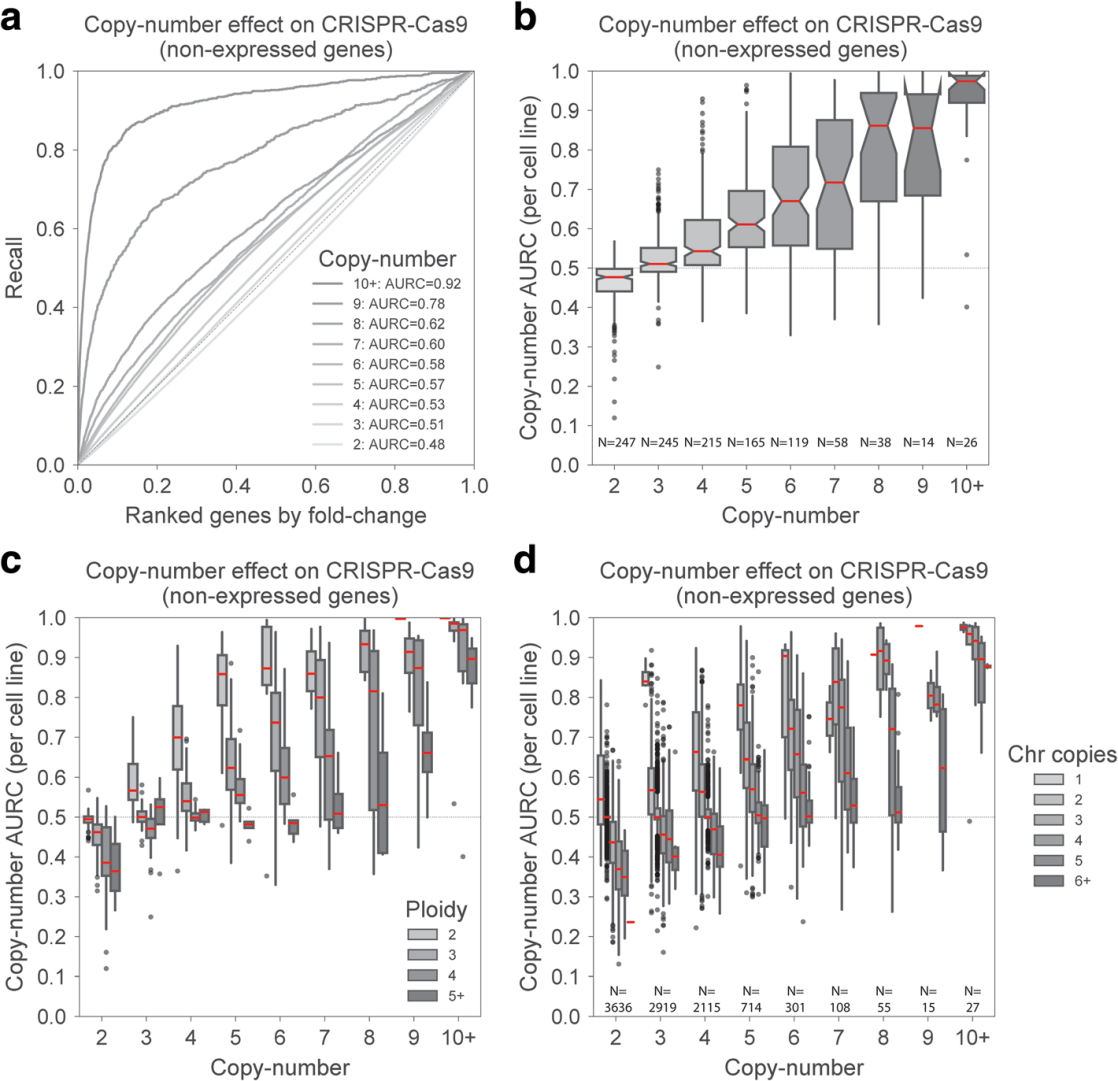
CRISPR-Cas9 screens and cell ploidy effect. **a** Enrichment of non-specific CRISPR-Cas9 LOF effects in non-expressed genes (RNA-seq RPKM < 1) grouped by their copy-number profile, performed across 250 cell lines. For each copy-number group, the recall curve is drawn and the area under the recall curve (AURC) is reported. *X*-axis shows the ranked gene level CRISPR-Cas9 fold changes, from negative to positive. **b** Boxplots of AURCs as in **a** but performed in each cell line independently. Each dot represents the AURC of the given gene copy-number in a specific cell line. **c** Similar to **b** but cell lines are grouped according to their ploidy status. **d** AURC of non-expressed genes estimated per chromosome in each cell line independently. Chromosomes were grouped according to their estimated number of copies. Boxplots represent 1.5 of the interquartile range

Consistent with previous findings [10, 20, 21], sgRNAs targeting copy-number amplified genes were among those with the strongest LOF effects in the screen, even when only considering genes which are not expressed (Fig. 1a). Importantly, the enrichments for LOF for each of the different copy-number levels varied considerably across cell lines, as can be attested by the large interquartile ranges of the distributions (Fig. 1b). This suggests that other factors, besides copy-number, contribute to non-specific LOF effects found in CRISPR-Cas9 data. Chromosomal aneuploidy is common in cancer, thus we investigated if cell ploidy across the heterogeneous panel of cell lines differentiates responses to CRISPR-Cas9. We observed that cells with higher ploidy display lower fitness reduction for sgRNAs targeting copy-number amplified regions (Fig. 1c). Diploid cells had significantly stronger LOF enrichments compared to tetraploid cells for all copy-number groups (Welch’s *t* test; *p* value < 0.05). Thus, the variation observed in each copy-number group defined in Fig. 1c can be in part accounted for by considering cell ploidy. Within the same cell line, different chromosomes can have different number of copies, thus we estimated the number of copies of each chromosome in each cell line and assessed if this was also related with non-specific CRISPR-Cas9 LOF effects. Consistent with the ploidy status, chromosomes with more copies display remarkably weaker gene-independent LOF effects (Fig. 1d). Overall, these results show that absolute copy-number profiles need to be analyzed together with cell ploidy, or chromosome copies, to model accurately the non-specific fitness reduction in CRISPR-Cas9 gene knockout experiments.

### Structural rearrangements are determinants of CRISPR-Cas9 LOF

**Fig. 2:**
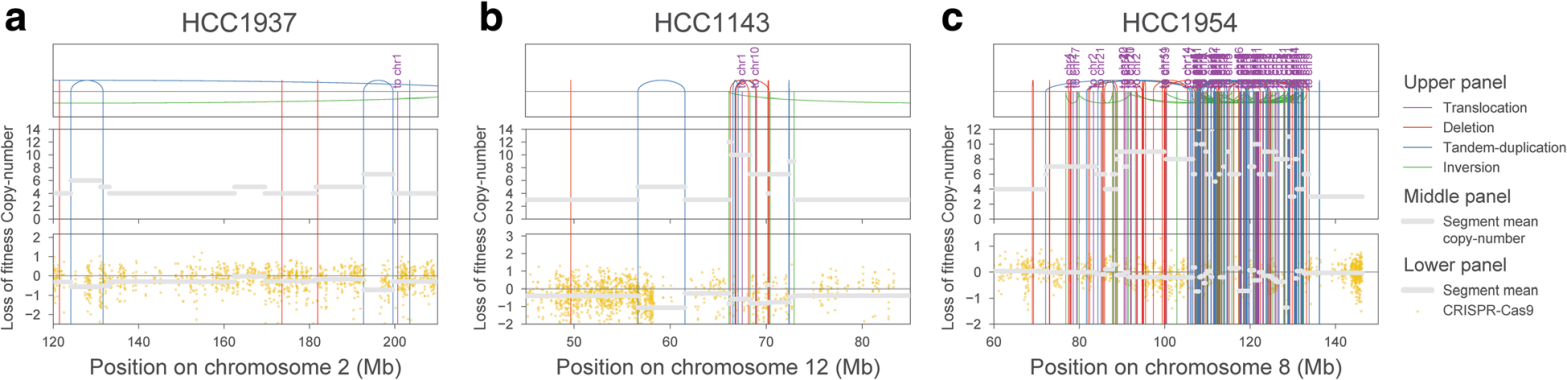
Structural variation impacts CRISPR-Cas9 response. **a**–**c** Representative examples of the strongest associations between SVs and CRISPR-Cas9 LOF. Structural rearrangements are mapped in the upper panel, in the middle panel copy-number levels are represented, and in the lower panel, CRISPR-Cas9 gene level fold changes are shown. SVs are colored with tandem duplications defined with blue lines, deletions with red lines, inversions in green, and chromosome translocations in purple. Average mean values for copy-number (middle panel) and CRISPR-Cas9 fold changes (lower panel) for each copy-number segment are represented as gray lines

Considering that SV is a common feature of cancer cells which can lead to copy-number change, we set to analyze their effect in CRISPR-Cas9 screens. WGS data from 4 breast cancer cell lines with matched normal were used to call somatic SVs, such as tandem duplications, translocations, deletions and inversions, using BRASS (BReakpoint AnalySiS) [26, 30]. Tandem duplications were the most frequent type of rearrangements across the 4 cell lines (Additional file 1: Figure S2a), recapitulating previous observations that this is a frequent event in breast cancers [26, 31]. We then examined a possible link between SV and CRISPR-Cas9 LOF effects. SVs were most informative of CRISPR-Cas9 response when accompanied by copy-number alterations, with LOF effects frequently falling within tandem duplications (Fig. 2a, b). Interestingly, complex patterns of SVs involving chromosomal translocations (Fig. 2c, Additional file 1: Figure S2b) were also visible and these overlapped with some of the strongest LOF responses observed. Not all copy-number amplifications, however, were associated with an increase in LOF (Additional file 1: Figure S2c), reflecting that different copy-number amplification mechanisms occur in cancer cells and these can lead to distinct CRISPR-Cas9 LOF effects. To disentangle some of the intrinsic SV complexity (e.g., nested rearrangements), we focused on tandem duplications and deletions that were supported by evidence of copy-number variation, specifically we searched for copy-number segments with start and end sites in close genomic proximity to the structural rearrangements. The number of events identified was low (*N* = 41 across all 4 cell lines, with a matching tolerance range of 10 Kb), and we found that tandem duplications have stronger, but not significant, LOF effects than deletions (Welch’s *t* test *p* value = 5.9e−2; Additional file 1: Figure S2d). Overall, these examples illustrate that SVs can determine gene-independent LOF effects in CRISPR-Cas9 experiments.

### Copy-number ratio improves the identification of CRISPR-Cas9 LOF effects

**Fig. 3:**
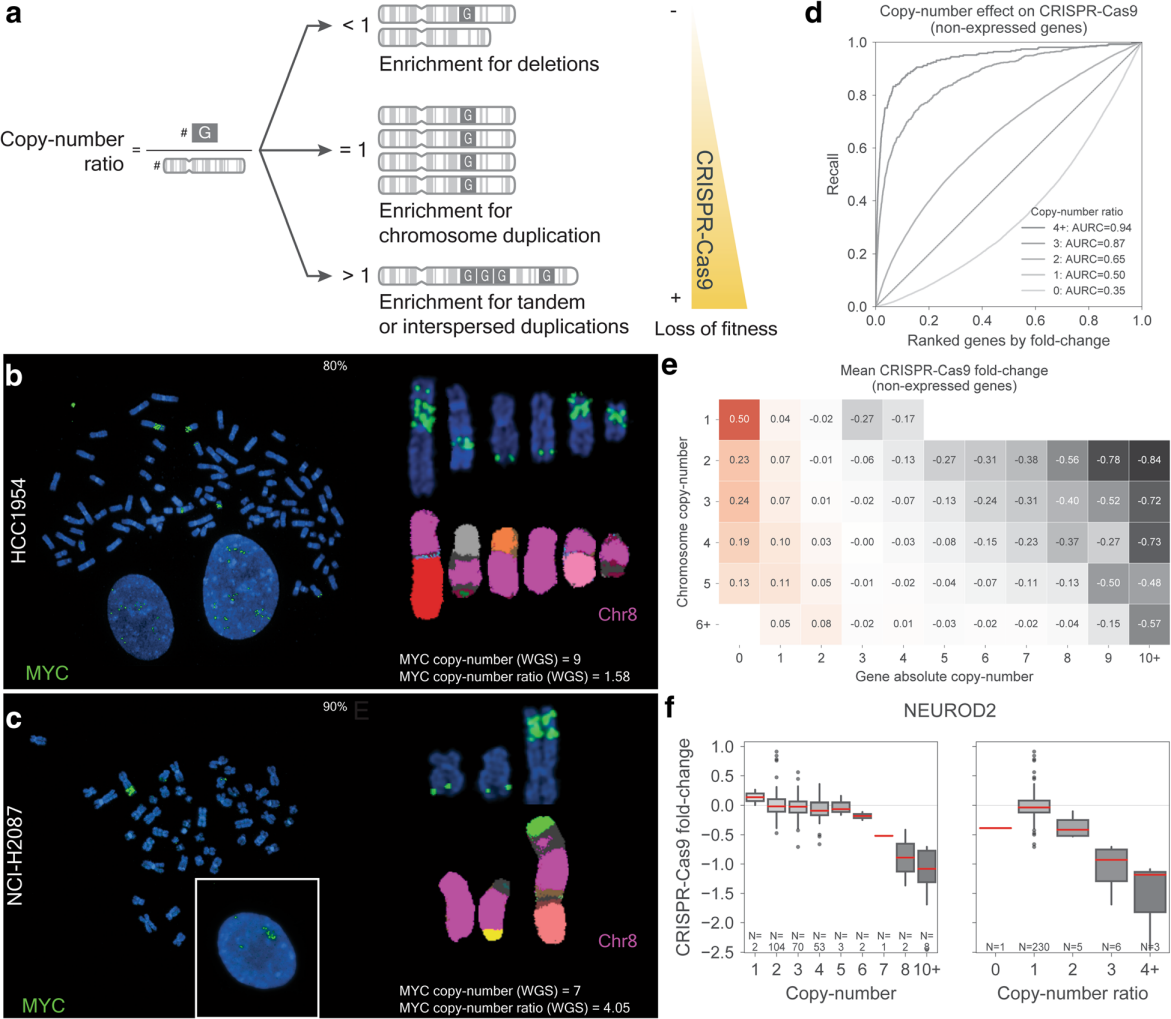
Gene copy-number ratio association with CRISPR-Cas9 loss of fitness effect. **a** Diagram of the different genomic rearrangements captured by the gene copy-number ratio and their potential effect in CRISPR-Cas9 response. **b** FISH of MYC amplifications (green signal) in HCC1954 tetraploid cell line. In the left panel, representative metaphase (80% of cells) with high MYC amplifications and low copy-number ratio. In the right panel, detailed view of the chromosomes containing MYC signal (upper) and the corresponding derivative chromosomes identified by M-FISH (lower). **c** Similar to **b**, FISH of MYC amplifications (green signal) in NCI-H2087 diploid cell line. In the left panel, representative metaphase (90% of cells) with high MYC copy-number ratio. For both cell lines, 10 cells were analyzed in the FISH experiments. **d** High copy-number ratios are enriched for strong CRISPR-Cas9 LOF effects. Recall curves of non-expressed genes grouped by their copy-number ratio profile across CRISPR-Cas9 fold changes. **e** Matrix of gene and chromosome copies representing the mean gene-level CRISPR-Cas9 fold change of non-expressed genes in the respective group. **f** Representative example of an amplified non-expressed gene with strong LOF effects associated with gene copy-number ratio (right panel) and to a lesser extent with absolute gene copy-number (left panel). CRISPR-Cas9 fold changes in **e** and **f** are scaled (known essential genes mean fold change = − 1)

Next, we set to comprehensively investigate the impact of structural rearrangements and ploidy in CRISPR-Cas9 experiments across different cancer types. To that end, we propose to normalize gene copy number by the number of chromosome copies, termed hereafter as gene copy-number ratio, on an individual cell line basis (Fig. 3a). Because WGS data to identify SVs were unavailable for most cell lines, this was performed across all 250 cell lines using copy-number profiles estimated with SNP6 arrays. The ratio encompasses three scenarios: a value (i) less than 1 represents a gene deletion, (ii) equal to 1 represents either a normal diploid chromosome with 2 copies of the gene or deletions/amplifications that are consistent between the gene and the chromosome, and (iii) greater than 1 represents genes that have been amplified more than the chromosome to which they map, likely representing tandem or interspersed duplications.

Consistent with our hypothesis, we confirmed that the stringent set of tandem duplications and deletions identified previously showed significantly higher copy-number ratios (median = 1.24) compared to deletions (median = 0.74) (Welch’s *t* test *p* value = 1.8e−3) (Additional file 1: Figure S2e). Higher copy-number ratios were significantly enriched for genes commonly amplified in tumors such as oncogenes, e.g., CCND1 and EGFR, conversely copy-number ratios smaller than one were enriched, but not significantly, for known tumor suppressors, e.g., CDKN2A and TP53 (Additional file 1: Figure S3a, Additional file 3: Table S2). We confirmed that high copy-number ratios represent strong focal tandem amplifications by performing fluorescence in situ hybridization (FISH) in two MYC amplified cell lines with distinct copy-number ratios (Fig. 3b, c). Moreover, chromosome copy-number estimations from SNP arrays were consistent with FISH karyotypes for the cell lines tested (Additional file 1: Figure S4a and S4b). Thus, gene copy-number ratio allows us to differentiate gene duplications that originate from whole chromosome/genome duplication from those arising from defined amplification events, such as tandem amplifications, which we hypothesize induces stronger CRISPR-Cas9 LOF effects.

Utilizing the copy-number ratio, we observed that non-expressed genes with copy-number ratios greater than 1 showed strikingly higher LOF effects (Fig. 3d). Ratios greater than or equal to 4 displayed among the strongest LOF effects captured in the screen, with non-expressed genes showing mean effects of 73.3% of those of known essential genes (Additional file 1: Figure S3b). The distribution of the copy-number ratios across all 250 cell lines was centered around 1, confirming that the vast majority of copy-number alterations originate from whole chromosome duplications (Additional file 1: Figure S3c). Notably, we observed thousands of occurrences of copy-number amplified genes with a copy-number ratio close to 1 which displayed no CRISPR-Cas9 LOF effects (Fig. 3e, Additional file 1: Figure S3c). As an example, among the frequently amplified and not expressed genes, neuronal differentiation 2 (NEUROD2) gene copy-number ratio recapitulated more clearly the LOF response than absolute copy number (Fig. 3f). Cell lines from multiple tumor types with 3, 4, 5 and 6 copies of NEUROD2 have very limited LOF effects, which could lead to incorrect correction of LOF effects in these cell lines if using approaches based on absolute copy number [22].

Taken together, our results indicate that non-specific LOF effects induced by targeting of copy-number amplified regions are enriched for tandem or interspersed duplicated regions, while copy-number amplifications originating from chromosome duplication have little to no effect. We cannot exclude that other complex structural rearrangements might also be captured by the gene copy-number ratio metric. Nonetheless, these observations have important implications for the analysis of CRISPR-Cas9 datasets, suggesting that correcting gene-independent LOF effects based on absolute copy number could, in many instances, lead to incorrect estimates.

### Crispy is a single sample copy-number correction tool for CRISPR-Cas9 screens

**Fig. 4:**
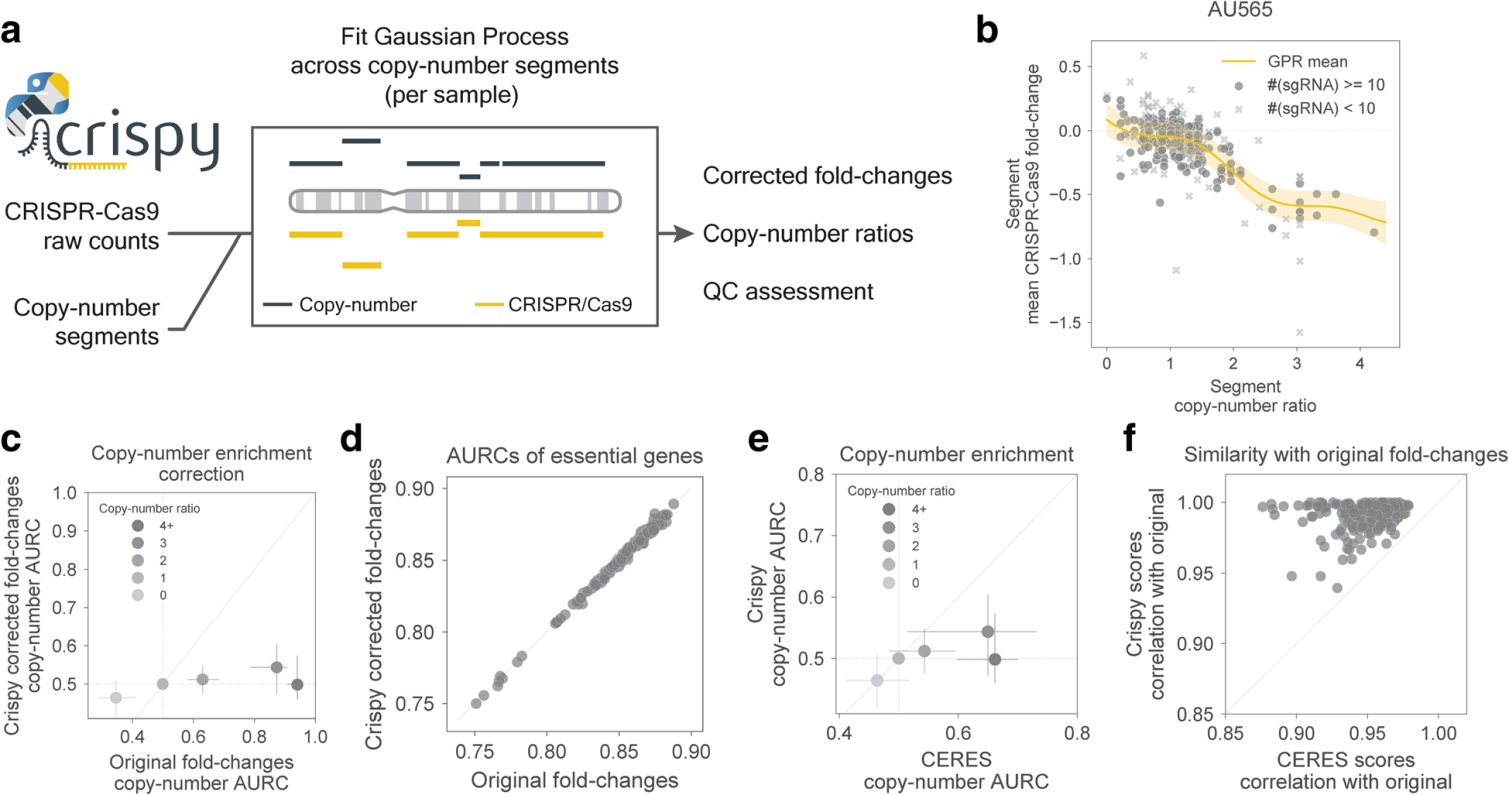
Crispy single sample correction at copy-number segment level. **a** Crispy pipeline taking as inputs sgRNA CRISPR-Cas9 raw-counts and copy-number segmentation. Gaussian process regression (GPR) is performed at the segment level on per sample basis. The tool outputs corrected fold changes and copy-number ratios and facilitates plotting functions for QC assessment. **b** Representative example of GPR fit on AU565 cell line. Yellow area represents the standard deviation estimated by the GPR. **c** Copy-number ratio AURCs obtained by ranking the original uncorrected CRISPR-Cas9 fold changes, *x*-axis, and the Crispy corrected fold changes, *y*-axis. **d** Recall of known essential genes before and after fold change correction. **e** Comparison between CERES and Crispy copy-number ratio AURCs of corrected fold changes. **f** Correlation of CERES and Crispy corrected fold changes with original uncorrected fold changes. Scatter plot in **c** and **e** represent the median AURCs and error bars the 25th and 75th percentile

To robustly account and correct for gene-independent copy-number LOF effects in CRISPR-Cas9 screens, we developed a Python module named Crispy (Fig. 4a). Crispy requires as input sgRNA CRISPR-Cas9 fold changes together with segment level copy-number measurements acquired, for example, from arrays (e.g., SNP6) or sequencing approaches (e.g., WGS). Gaussian Process regressions are used to model the non-linear associations between the copy-number ratio and the impact on CRISPR-Cas9 fold changes. Fitting is performed at the segment level, whereby segments identified by copy-number segmentation algorithms are overlapped with CRISPR-Cas9 sgRNAs, and averaged fold changes for the segments are calculated. Segment copy-number ratios, estimated similarly to gene copy-number ratios, are then used to model the segment mean fold changes (Fig. 4b). Segments containing less than 10 sgRNAs are not considered for the fitting to limit the impact of potential outliers arising from a low number of measurements. Contrary to methods that need to be trained across panels of different cell lines, Crispy is trained on a per sample basis to consider cell-specific effects such as ploidy. Of note, Crispy takes into consideration that high copy-number amplifications might have no impact if arising from whole chromosome amplifications, avoiding potential miscorrection of CRISPR-Cas9 fold changes. We generated Crispy corrected fold changes across the 250 cell lines, these showed strong attenuation of the copy-number ratio bias compared to the original fold changes (Fig. 4c), while preserving the recall capacity of known essential genes (Fig. 4d).

Next, we benchmarked our approach against another copy-number correction tool, CERES [22], which contrary to Crispy performs a sgRNA level correction across multiple samples. We observed that Crispy provides a small but consistent improvement, reducing the median AURCs across the different copy-number ratios (Fig. 4e, Additional file 1: Figure S5a and S5b). Unlike CERES, Crispy does not boost the recall of known essential genes compared to the original fold changes (Additional file 1: Figure S5c). These improvements are more prevalent in cell lines that display weaker correlation between replicates (Additional file 1: Figure S5d), likely due to CERES modeling sgRNAs LOF as a shared effect across multiple cell lines, and therefore borrowing information from other samples. Consistent with this, Crispy corrected fold changes were more similar to the original fold changes (Fig. 4f), indicating that it effectively preserves the underlying LOF effect while correcting for bias due to unbalanced structural alterations.

In summary, Crispy is an open-source Python tool that can be used to correct in a supervised way CRISPR-Cas9 gene independent copy-number LOF effects on per sample basis using segment level copy-number ratios.

## Discussion

In this study, we demonstrate that copy-number amplifications lead to non-specific CRISPR-Cas9 LOF effects if originating from structural rearrangements, in particular tandem or interspersed duplications. In contrast, little or no impact is observed from gene amplifications associated with increased cell ploidy. Therefore, gene independent LOF effects seem to be cell line specific. We devised a gene copy-number ratio metric, normalized by chromosome copy-number, that improves the ability to classify which copy-number amplifications will result in a LOF bias in CRISPR-Cas9 experiments. Notably, our findings were recapitulated when considering only non-expressed genes, emphasizing that the LOF bias is not due to a potential biological function of the genes. Combining the copy-number ratios, WGS, and FISH experiments, we found that tandem duplications are amongst the most frequent SVs associated with CRISPR-Cas9 non-specific deleterious effects. Based on these observations, we developed a computational method, Crispy, to perform on a single sample basis the correction of CRISPR-Cas9 LOF effects due to targeting copy-number amplified regions. Crispy corrected fold changes retained high correlation with the original fold changes, preserved the ability to recall known essential genes, and improved on methods designed to correct these effects across different samples.

While this work furthers our understanding of the implication of SV in interpreting CRISPR-Cas9 screens several limitations remain. Due to the complexity of structural rearrangements we cannot exclude that other events might also play a role. For example, extrachromosomal DNAs (ecDNA) have been found to be widespread in cancer [32, 33] and are tandem duplicated rich DNA sequences, although we have no evidence of ecDNAs in the cell lines analyzed with FISH. Furthermore, we also observed that complex structural rearrangements involving multiple unbalanced chromosomal translocations overlap with some of the strongest LOF effects in the screens. This illustrates the complexity and the limitations of interpreting accurately the efficacy of CRISPR-Cas9 reagents that target a locus that is structurally rearranged.

Structural rearrangements are amongst the most common alterations in cancer [25] indicating that our findings are of general importance when designing and interpreting CRISPR-Cas9 experiments in cancer cells. Specifically, targeting genes that reside within tandem duplicated or highly rearranged and unbalanced regions, whether knocking out individual genes, performing genetic screens using a library of sgRNAs, or performing specific gene edits, will lead to strong non-specific LOF effects. While Crispy can correct for this effect in CRISPR-Cas9 sgRNA library screens, for many studies sufficient data to robustly train models to correct these effects is unlikely to be available, for example the common scenario of a single gene knockout in an individual cell line. In these instances, information about gene copy-number, cell line ploidy and ideally SV information should be incorporated to guide interpretation of LOF effects. Furthermore, the use of an orthogonal technology such as RNAi or CRISPR interference to corroborate results is advisable. For CRISPR-Cas9 sgRNA library screens where reliable copy number data are unavailable, an unsupervised LOF correction method such as CRISPRcleanR [23] can be used. We expect that the bias in CRISPR-Cas9 data described here is a general phenomenon and consequently will be observed in other cancer cell models such as patient-derived xenografts and organoids, and are potentially also present in other types of CRISPR-Cas-based systems that introduce DNA double-strand breaks [34, 35].

## Conclusions

CRISPR-Cas9 targeting of structurally rearranged regions, in particular tandem or interspersed amplifications, is detrimental to cellular fitness in a gene independent manner. Targeting amplifications caused by whole chromosomal duplications has little to no impact on fitness. Crispy is a computational tool that accounts for this effect in screening data by correcting copy-number bias on a per sample basis. Our findings have implications for the design, analysis, and interpretation of CRISPR-Cas9-based methods for commonly used approaches such as gene knockouts, sgRNA library screens and targeted gene-editing.

## Materials and methods

### Processing of CRISPR-Cas9, SNP6, and RNA-seq samples

Publicly available CRISPR-Cas9, BROAD DepMap 18Q3, drop-out screens across 250 cell lines was utilized to assess the loss of fitness (LOF) impact of knocking-out 17,328 genes [22, 27]. Raw sequence counts of each sgRNA were downloaded and corrected by library size in each sample. Non-targeting plasmid control sample was used and sgRNAs with lower than 30 counts were discarded. Log2 sgRNA fold changes were estimated between samples and the plasmid control. Gene level estimates of the fold changes were calculated by averaging all mapping sgRNA fold changes. Single nucleotide polymorphism (SNP) array hybridization using the Affymetrix SNP6.0 platform was performed according to Affymetrix protocols. Segment copy-number variants were obtained using PICNIC [36] as previously described [29]. RNA-seq experiments for CRISPR-Cas9 profiled cell lines were assembled from multiple data-sets [37]. To minimize technical bias, all samples were processed with the same pipeline, iRAP [38], to obtain raw counts. Genes with Reads Per Kilobase per Million (RPKM) with zero counts were termed as non-expressed in the particular sample. Non-expressed genes were defined as those with a RPKM lower than 1.

### Chromosome harvest and fluorescence in situ hybridization (FISH)

Metaphase chromosomes were harvested from the cancer cell lines after incubation with 0.05 g/ml of colcemide (Thermo-Fisher) for 2–3 h. Subsequently, cells were treated with a buffered hypotonic solution (0.4% KCl in 10 mM HEPES, pH 7.4) for 8–12 min at 37 °C and fixed with 4:1(*v*/v) methanol: glacial fixative. The human fosmid clone WI2-1694H13 was labeled with green-dUTP as described in [32]. Human 24 color FISH (M-FISH) probe preparation and slides treatments followed [39] with slight modifications. Freshly-prepared metaphase slides were immersed in acetone for 10 min and then baked at 62 C for 1 h. Slides were denatured in an alkaline denaturation solution (0.5 M NaOH, 1.5 M NaCl, Sigma-Aldrich) for 9–10 min. Metaphases were examined with a Zeiss AxioIamger D1 fluorescence microscope. FISH images were captured using the SmartCapture software (Digital Scientific UK) and karyotyped using the SmartType Karyotyper software (Digital Scientific UK). Ten metaphases for each sample were analyzed by M-FISH.

### Whole-genome sequencing

DNA of 4 cancer cell lines and 4 EBV derived matched normal cell lines were obtained and sequenced with massively parallel Illumina sequencing technology (EGAD00001004124) and aligned to the human reference genome GRCh37 using Burrows-Wheeler Aligner (v0.5.9) [40]. Average sequence coverage was 43-fold for cancer cell lines and 42-fold for matched normal. Somatic structural rearrangements were identified by providing aligned bam files to BRASS (BReakpoint AnalySiS) (https://github.com/cancerit/BRASS/). BRASS calls structural variations via assembly of discordant paired-end reads.

Identification of structural rearrangements overlapping with copy-number segments was limited to tandem duplications and deletions identified with BRASS. To increase confidence in the SVs found, a BRASS assembly score was required for the tandem duplication or deletion to be considered. Then for each cell line, the tandem duplications and deletions were searched against all copy-number segments (identified with PICNIC) to find those SVs and segments for which start and end sites overlapped. A mismatch tolerance range of 10 Kbp was used for both start and end sites.

### Crispy, single sample method to correct copy-number gene-independent effects in CRISPR-Cas9 screens

Crispy is a Python tool to model the copy-number impact on LOF effects in CRISPR-Cas9 on a per sample basis. For each sample the required inputs are (i) the sgRNA raw-counts together with their targeting genomic information and (ii) the copy-number segmentation output, typically a BED file containing the segment mapping chromosome, start and end genomic positions and absolute copy-number. Segments and sgRNAs are intersected using BEDtools (v2.27.1) [41] and pybedtools (v0.7.10) [42]. For each segment defined by the copy-number segmentation algorithm two metrics are computed: (i) a copy-number ratio, i.e., segment copy-number divided by the estimated chromosome copy-number; and (ii) a mean CRISPR-Cas9 fold change of all the sgRNAs that overlap with the segment. Gaussian Processes regression implemented on scikit-learn Python module (v0.19.1) [43] is used to model the non-linear effects between the segment copy-number ratio and the CRISPR-Cas9 fold changes. Specifically, a squared-exponential kernel (RBF) with a length scale (*ө*) hyperparameter varying between 1e−5 and 10 is used. A constant (*σ*) and noise (ψ) kernels are also added:

1 $$ K\left(x,{x}^{'}\right)={\sigma}^2\exp \left(-{\left(x-{x}^{'}\right)}^2/\left(2{\uptheta}^2\right)\right)+\psi $$

where ө determines the length of the waves and *σ* defines the average distance from the mean. Default configurations of scikit-learn Gaussian regression are used except n_restarts_optimizer that is set to 3, to initialize the optimization procedure multiple times. The kernel defined in [1] is fitted independently for each sample. This guarantees that CRISPR-Cas9 global effects that are sample specific are captured automatically by the defined kernel. After training, the CRISPR-Cas9 corrected fold changes are obtained by subtracting from the original fold changes the predicted bias from the inputted segment copy-number ratios.

### Gene copy-number ratios

Gene copy-number ratios, i.e., number of absolute gene copies divided by the number of copies of the respective chromosome, are calculated for all genes covered by the CRISPR screens and SNP6 arrays. Gene and chromosome absolute copy-number values are estimated by taking the copy-number weighted mean of all the mapping segments weighted by their size.

To verify that high copy-number ratios represent focal chromosome amplifications, we applied FISH and probed the location of the frequently and highly amplified oncogene MYC. We chose 2 cell lines (HCC1954 and NCI-H2087) with high MYC absolute copy-number (9 and 7, respectively) but discordant copy-number ratios (1.58 and 4.05, respectively) due to different ploidy. A control triploid cell line (LS1034) with diploid MYC and copy-number ratio of 1 was analyzed and corroborated our prediction that chromosome 8 is mostly diploid and contains 2 copies of MYC (Additional file 1: Figure S4a).

### Code availability

Crispy is a Python module (https://github.com/EmanuelGoncalves/crispy), and its code and the source code for the analysis in this manuscript are distributed under the open-source 3-Clause BSD License. To facilitate usability and adaptation, Crispy can be easily installed through the commonly used PyPI repository (https://pypi.org/project/cy/) and instructions are provided.

## Additional files

Additional file 1: **Figure S1.** CRISPR data overview and quality assessment. **Figure S2.** Structural rearrangements association with CRISPR-Cas9 response. **Figure S3.** Gene copy-number ratios. **Figure S4.** FISH and M-FISH experiments. **Figure S5.** Crispy benchmark against CERES. (PDF 909 kb)

Additional file 2: **Table S1.** List of cancer cell lines included in the study. (XLSX 30 kb)

Additional file 3: **Table S2.** Mean gene copy-number ratios of cancer associated genes across the cancer cell line panel. (XLSX 14 kb)
