# Supplementary material for: Structural rearrangements generate cell-specific, gene-independent CRISPR-Cas9 loss of fitness effects
Source: Genome Biol. 2019 Feb 5;20:27. doi: 10.1186/s13059-019-1637-z (PMC6362594; doi:10.1186/s13059-019-1637-z)
Supplement: Supplementary file 1 — Figure S1. CRISPR data overview and quality assessment. Figure S2. Structural rearrangements association with CRISPR-Cas9 response. Figure S3. Gene copy-number ratios. Figure S4. FISH and M-FISH experiments. Figure S5. Crispy benchmark against CERES. (PDF 909 kb) [file 13059_2019_1637_MOESM1_ESM.pdf]

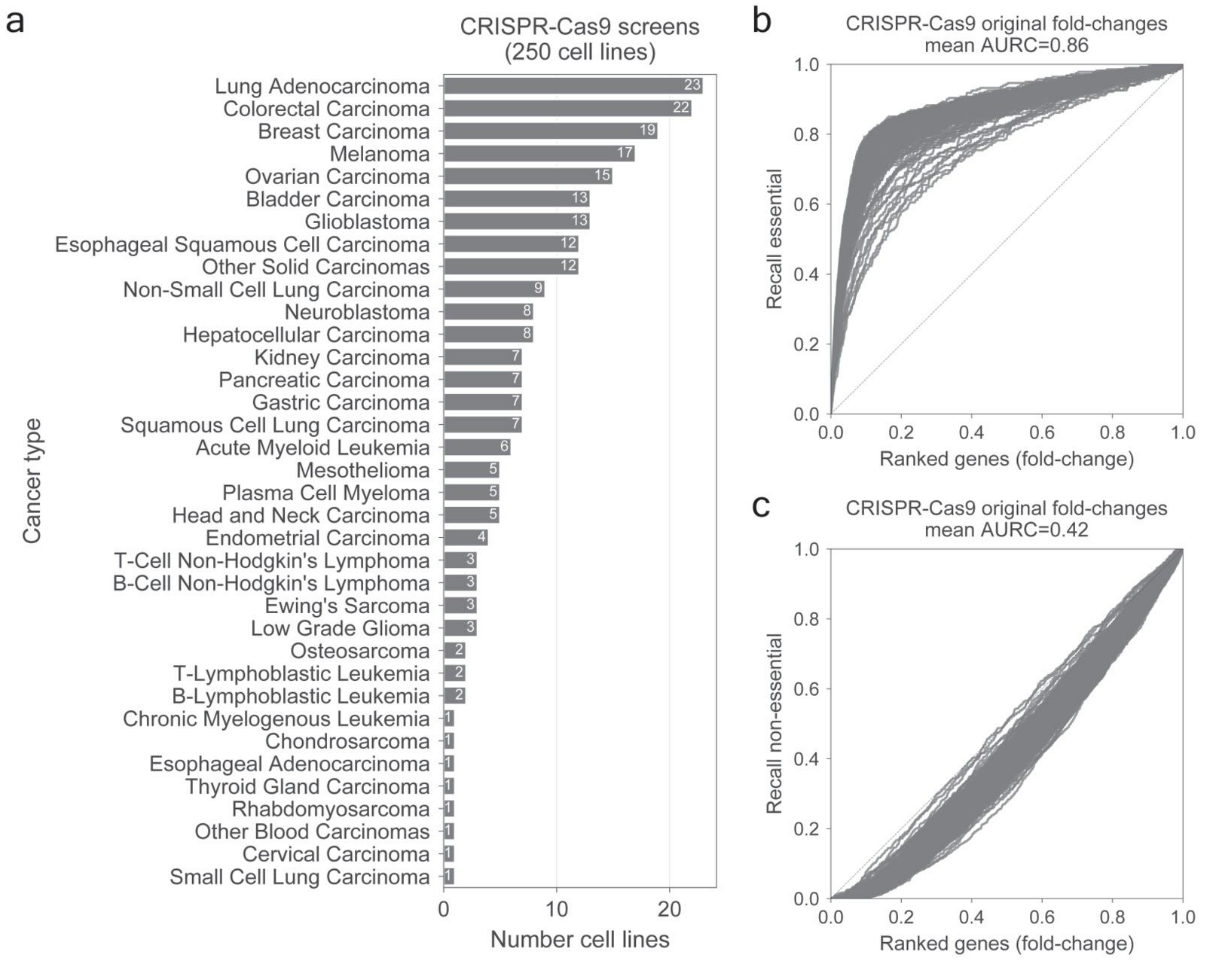

Figure S1. CRISPR data overview and quality assessment. *a) Number of cell lines screened per cancer type. Recall curves of previously defined b) core-essential, and c) non-essential genes [15]. X-axis represents the ranked CRISPR-Cas9 gene-level fold-changes from negative to positive. AURC of each curve was estimated and the mean is reported.*

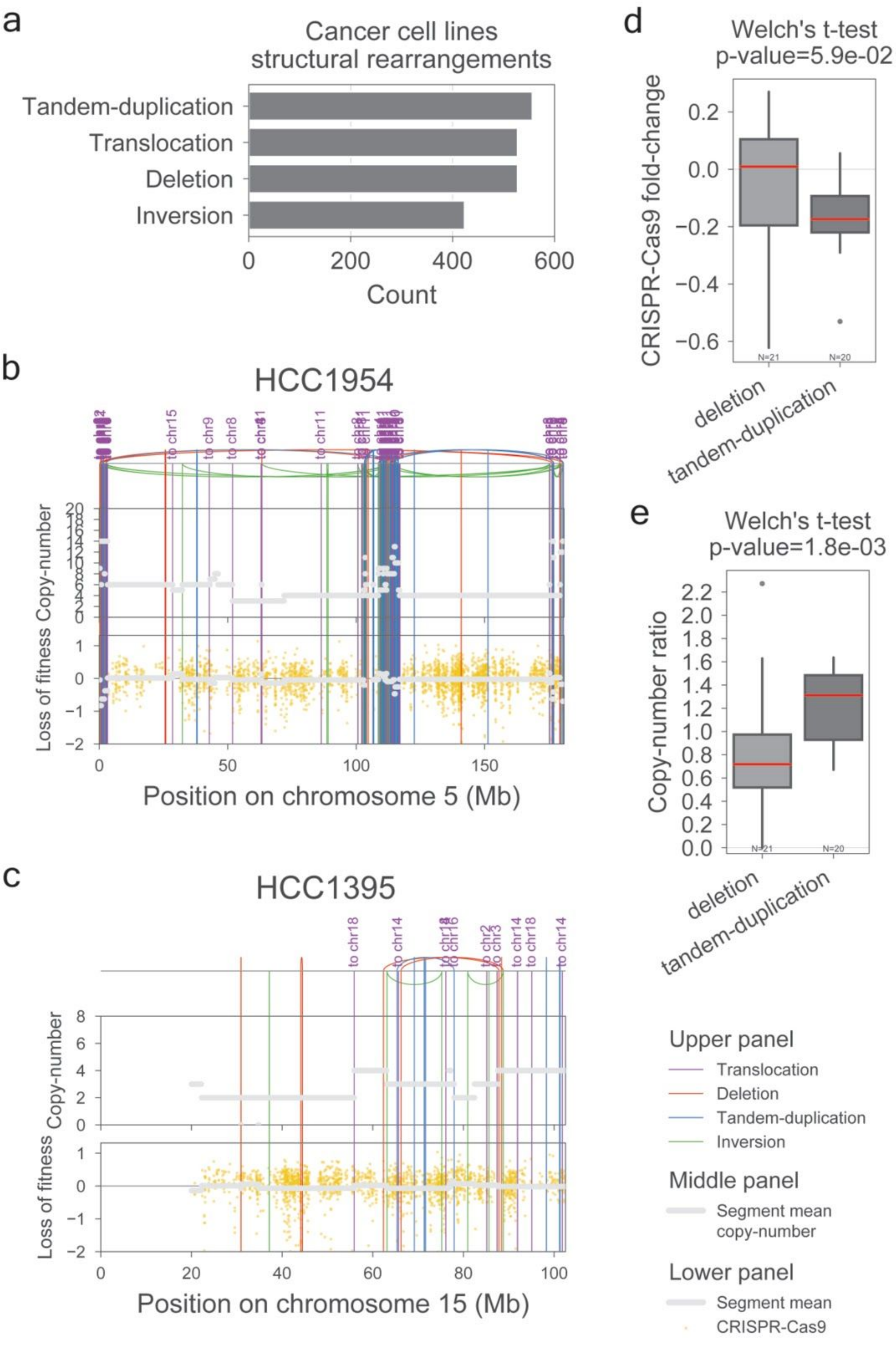

a
Cancer cell lines
structural rearrangements
Tandem-duplication
Translocation
Deletion
Inversion
0
200
400
600
Count
b
HCC1954
Copy-number
Loss of fitness
Position on chromosome 5 (Mb)
c
HCC1395
to chr18
to chr14
to chr2
to chr3
Copy-number
Loss of fitness
Position on chromosome 15 (Mb)
d
Welch's t-test
p-value=5.9e-02
CRISPR-Cas9 fold-change
N=21
N=20
deletion
tandem-duplication
e
Welch's t-test
p-value=1.8e-03
Copy-number ratio
N=21
N=20
deletion
tandem-duplication
Upper panel
Translocation
Deletion
Tandem-duplication
Inversion
Middle panel
Segment mean
copy-number
Lower panel
Segment mean
CRISPR-Cas9

Figure S2. Structural rearrangements association with CRISPR-Cas9 response. *a) Count of somatic structural rearrangements identified in 4 breast cancer cell lines. b) Association between SVs and CRISPR-Cas9 loss of fitness involving complex patterns of SVs containing a high number of chromosomal translocations aligned with copy-number changes. c) Representative example of lack of non-specific loss of fitness effects in copy-number amplified regions. d) Mean CRISPR-Cas9 fold-changes and e) copy-number ratios for BRASS identified deletions and tandem duplications overlapping with copy-number segments (10 Kbp match tolerance range for start and end sites).*

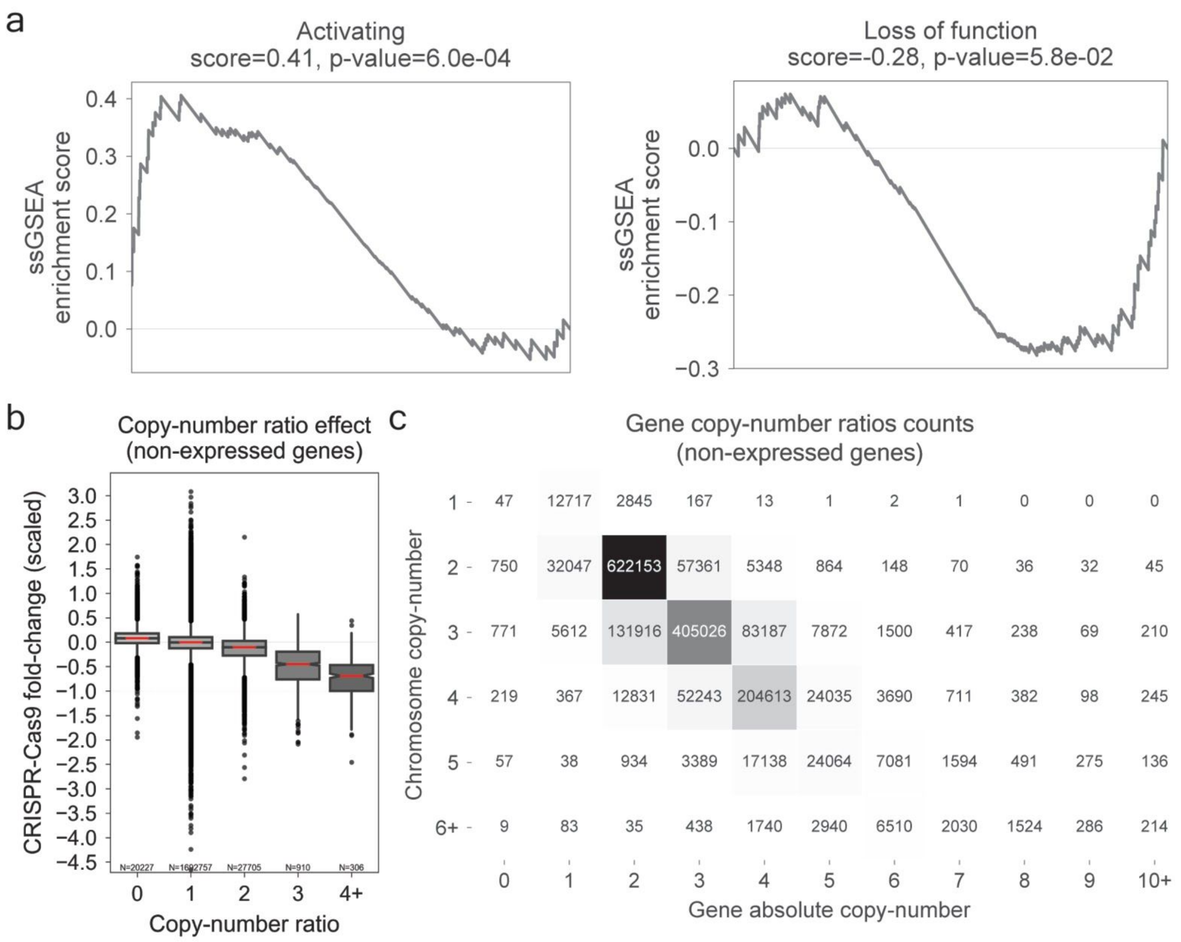

Figure S3. Gene copy-number ratios. *a) Single-sample GSEA enrichment for known oncogenes (Activating) and tumour-suppressor (Loss of function) genes. Empirical p-values were estimated by randomising the gene-sets 10,000 times. b) CRISPR-Cas9 scaled fold-changes (essential genes = -1) of non-expressed genes grouped according to their copy-number ratio. c) Gene copy-number ratios count across gene and chromosome copies.*

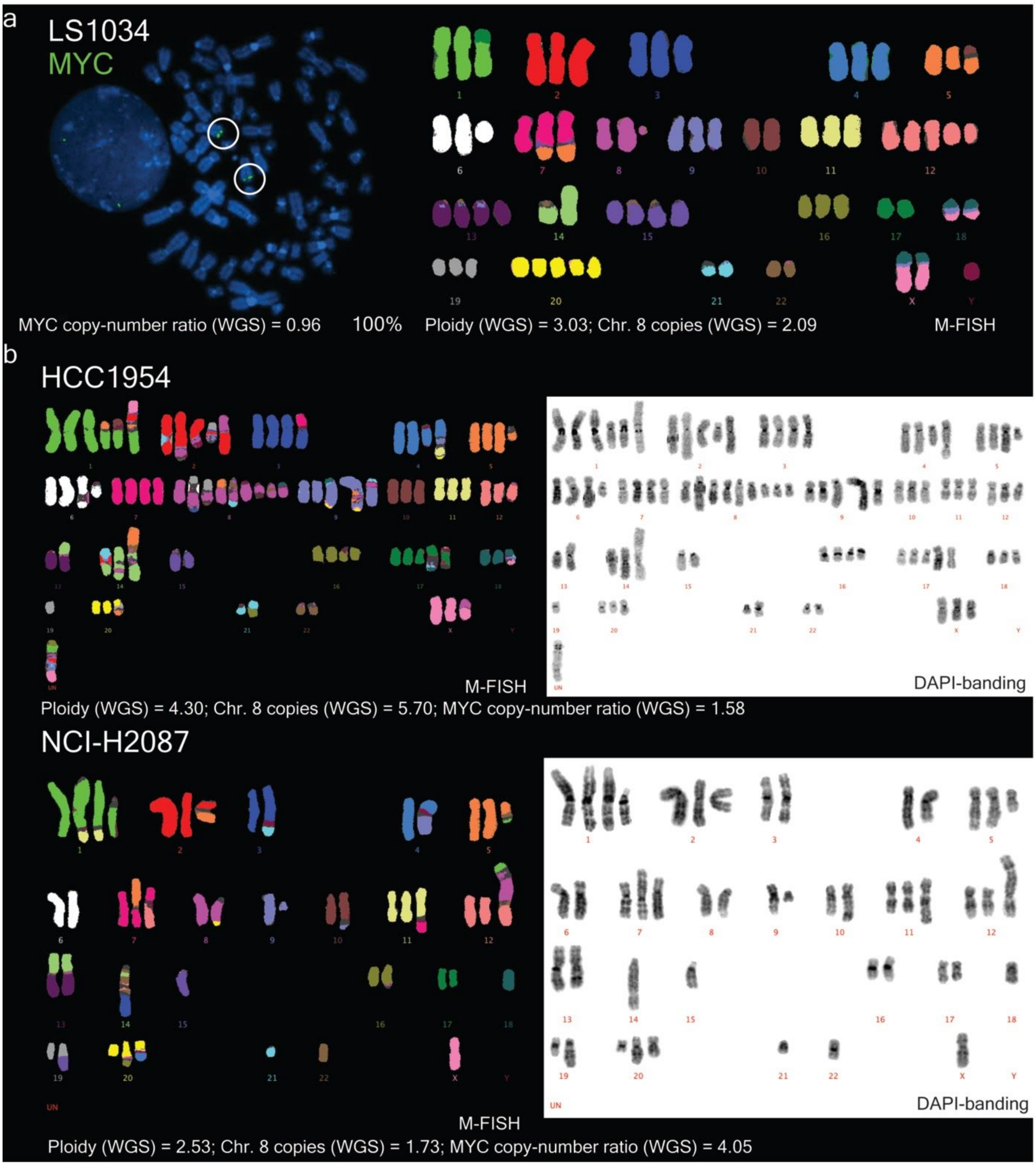

Figure S4. FISH and M-FISH experiments. *a) FISH with green fluorescent MYC probe in triploid cell line with 2 MYC copies (left). M-FISH representative karyotype (right). 10 cells were used and the karyotype was observed in 100% of the cells. b) M-FISH representative karyotypes (10 cells) of two MYC amplified cell lines. MYC copy-number ratio, ploidy and chromosome copy-number were estimated from WGS.*

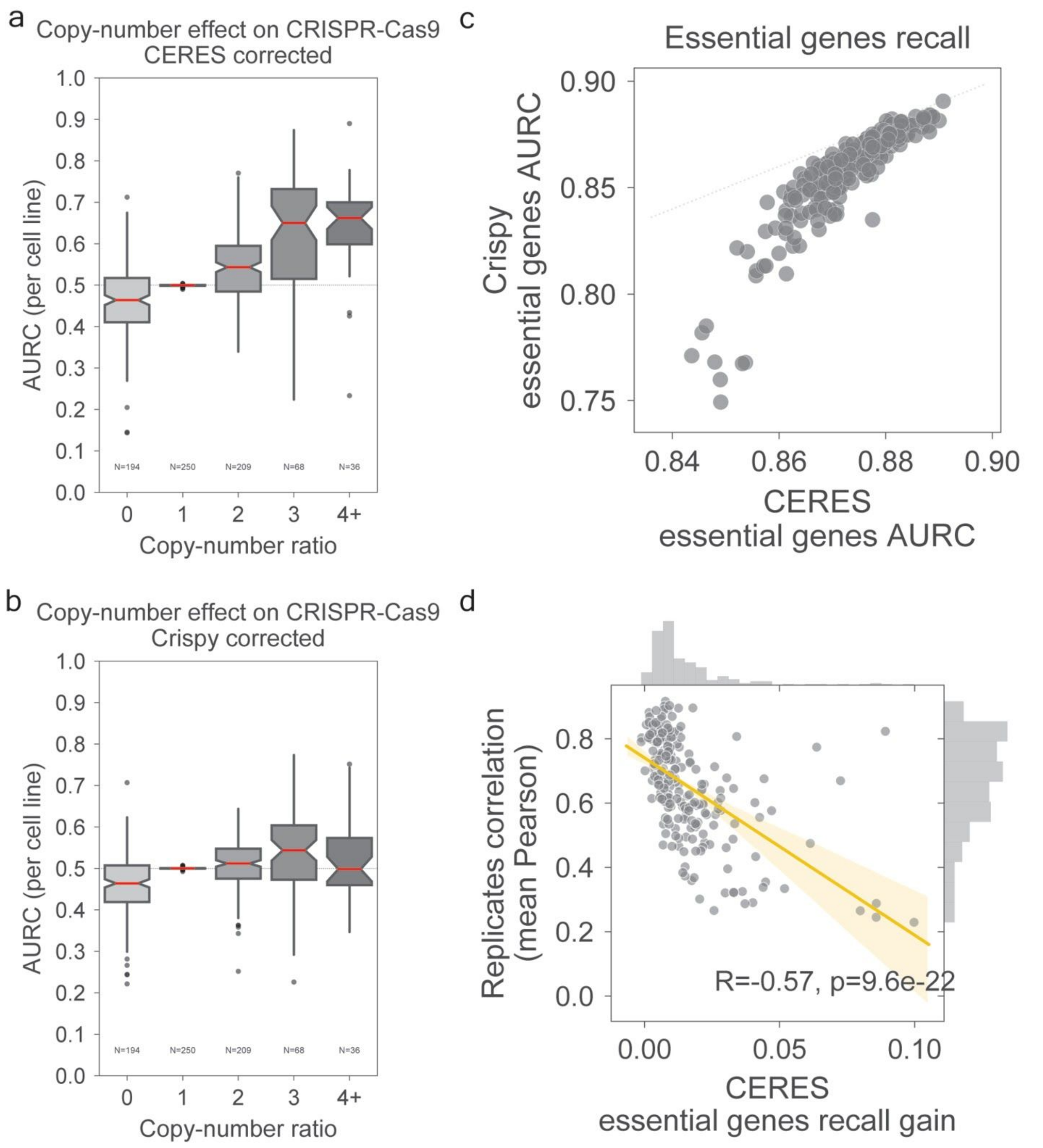

Figure S5. Crispy benchmark against CERES. *a) Gene-level AURCs per cell line for each copy-number ratio threshold using CERES corrected CRISPR-Cas9 fold-changes. b) Similar to a, using Crispy corrected fold-changes instead. c) Essential genes [15] recall comparison between CERES and Crispy. d) CERES recall gain compared to Crispy versus correlation of cell line replicates.*
